# Portable thermal scanners to detect and monitor small endotherms: A comparative assessment of available equipment to guide practitioners

**DOI:** 10.1002/ece3.10331

**Published:** 2023-07-21

**Authors:** Finella M. E. Dawlings, Claire Mackay, Morgan Humphrey, William F. Mitchell, Karina J. Sorrell, Sonia Sanchez, Benjamin M. Viola, Rohan H. Clarke

**Affiliations:** ^1^ School of Biological Sciences Monash University Clayton Victoria Australia

**Keywords:** detection distance, detection rate, endotherm, nocturnal, spotlighting, thermal

## Abstract

Detection is essential to studying and monitoring wild animals; however, detection is challenging for small endotherms that are nocturnal or best detected at night. Techniques such as trapping or spotlighting disturb focal species, and the effectiveness of spotlighting can be limited for cryptic species, resulting in low detection rates that hinder our ability to monitor and study some endotherms at night. Thermal scanners detect infrared wavelengths not otherwise visible to humans. With improvements in equipment size and cost, thermal scanners have emerged as a valuable tool for passive detection of endotherms. Here we seek to provide objective guidance on thermal tool selection to practitioners who wish to adopt such tools to detect and monitor small endotherms. We compared the efficacy of three handheld thermal scanners (of varying resolutions) and a traditional spotlight for detecting small, cryptic endotherms at night. Random arrays of artificially heated small bird models (representing small, cryptic endotherms) were established along transects in native grasslands that support a range of small threatened endotherms, including the Critically Endangered Plains‐wanderer (*Pedionomus torquatus*). Transects were independently surveyed by five observers, blind to model locations and model density. Performance measures representing detection capability were assessed for all devices, and usability of each device was assessed with a survey completed by all observers. Detection rates, detection distances, and survey accuracy were greater for thermal scanners with resolutions of 320 × 240 and 640 × 480 than for the spotlight. A low‐resolution thermal scope (160 × 120) performed poorly for all performance measures. There was a consensus among users that a video camera‐style thermal scanner was most comfortable to hold and view while traversing the transect, as opposed to thermal scopes where users look directly through the lens. These results suggest that high‐resolution thermal scanners (≥320 × 240) provide improved detection capabilities compared to traditional spotlights. Higher detection rates provide opportunities for detecting and monitoring small endotherms at night where this was once difficult or impossible.

## Graphical abstract

We compared detection capabilities of three handheld thermal scanners and a traditional spotlight for detecting small, cryptic endotherms at night by establishing random arrays of artificially heated small bird models along transects, which were surveyed with each device by five observers blind to model locations and density. High‐resolution thermal scanners (≥320 × 240) provide improved detection rates, detection distances, and survey accuracy compared to traditional spotlights. This study provides objective guidance on thermal tool selection for practitioners and shows that thermal scanners present opportunities to detect small endotherms at night where this was once difficult or impossible.

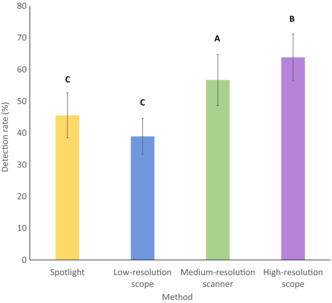

## 1 INTRODUCTION

Detecting wild animals is essential to understanding their ecology and conducting population monitoring. Detection is particularly challenging for small endotherms, because they are often nocturnal, display avoidance behaviors, are camouflaged, or occur in low densities (Chadès et al., 2008; Jones, 2011; MacKenzie et al., 2005; Witmer, 2005). Many small endotherms are best detected at night, and monitoring has traditionally been achieved with pitfall, cage, or camera traps—all of which are time‐consuming to set and retrieve and are disruptive to the focal species and/or its environment. Spotlight searches are a more passive approach, but rely on visual detection, favoring species that are non‐stationary or have bright reflective eyeshine. As such, spotlighting may be limited for cryptic species, leading to low detection rates hindering our ability to study and monitor cryptic endotherms at night. Thermal scanners offer a rapidly emerging, passive approach to detecting endotherms at night (Cilulko et al., 2013; Wilson & Delahay, 2001).

Thermal scanners have emerged as a non‐invasive tool for efficient detection of endotherms (Cilulko et al., 2013; Mitchell & Clarke, 2019). Thermal scanners generate a real‐time digital image of heat emitted from objects through the detection of infrared radiation. This facilitates detection of endotherms through body heat alone, regardless of movement, eyeshine, or cryptic coloration (McCafferty, 2007). A wide range of portable thermal scanners have been developed, with varying resolutions and price points. For example, thermal scanners are currently available as handheld video cameras, handheld scopes, road vehicle‐ and vessel‐mounted scanners, as well as drone‐ and manned aircraft‐mounted cameras (Donlon et al., 2014; Hovinen et al., 2008; Karp, 2020; McCafferty, 2007; Mutalib et al., 2019; Nugent, 2019). Thermal scanners have performed well on endotherms with a wide range of body sizes ranging from Brown Hare leverets (*Lepus europaeus*) to Moose (*Alces alces*; Augusteyn et al., 2020; Burke, Rashman, Longmore, et al., 2019; Cilulko et al., 2013; Gooday et al., 2018; Karp, 2020; Mitchell & Clarke, 2019). Limitations of thermal scanners as a wildlife monitoring tool can include reduced visibility in dense vegetation, delays in calibration time, inadequate resolutions and field of views, and high cost of equipment purchase (Burke, Rashman, Wich, et al., 2019; Hanson et al., 2014; Mutalib et al., 2019).

Here we directly compare multiple thermal scanner models in conjunction with a comparison to traditional spotlighting as tools for detecting small endotherms at night under field settings. Thermal scanners have a relatively high investment cost and are available in a wide range of models with varying applicability between different species and environments. As such, we sought to determine the ‘best practice’ for wildlife managers seeking to detect and monitor small endotherms at night.

**FIGURE 1:**
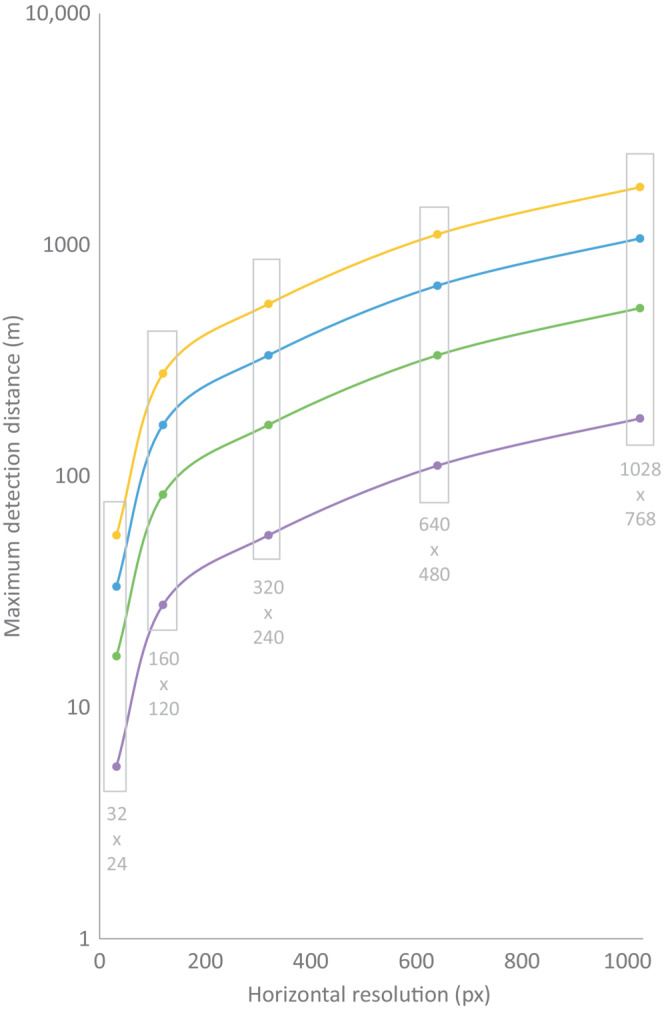
Theoretical maximum detection distance (log scale) obtained from handheld thermal scanners for four different animal ‘body sizes’ representative of common endotherms; 100 cm × 100 cm (yellow line; e.g. deer), 60 cm × 60 cm (blue line; e.g. fox), 30 cm × 30 cm (green line; e.g. possum) and 10 cm × 10 cm (purple line; e.g., mouse or Plains‐wanderer). Horizontal resolution of thermal scanner is displayed on *x*‐axis. Distances were calculated based on ‘Detection’ category of Johnson's Criteria and standard 22° horizontal field of view (Johnson, 1985). Gray boxes show five standard resolutions of thermal scanners.

We used thermal scanners to survey simulated populations of Plains‐wanderers (*Pedionomus torquatus*), a small Critically Endangered bird traditionally detected at night via spotlighting, in native grasslands in south‐eastern Australia. We tested the efficacy of a handheld video camera‐style thermal scanner and two thermal scopes for detecting small, cryptic endotherms at night in open habitats. We compared detection distances, detection rates, precision, accuracy, and usability of each device. Technical specifications of thermal scanner models are expected to influence detectability of endotherms with an expectation that detection distance will increase with thermal scanner resolution and animal size (Johnson, 1985; Figure 1; Appendix 1—Figure A1).

## 2 MATERIALS AND METHODS

### 2.1 Study site

Sampling was conducted on native grasslands in Victoria, Australia, which are flat, naturally treeless, and representative of our model species' native habitat (Nugent et al., 2022). Two transects were surveyed in the Western Grasslands, Werribee, Victoria, in October 2019, and four at Terrick Terrick National Park, Victoria in June 2020. Surveys were conducted after last light (18:24–24:00 h) in minimal rain (0–0.6 mm/h), low wind (<15 km/h), moderate‐high relative humidity (65%–94%) and cool to mild temperatures (minimum −0.1 to 8.5°C; Bureau of Meteorology, 2019, 2020).

### 2.2 Data collection

**FIGURE 2:**
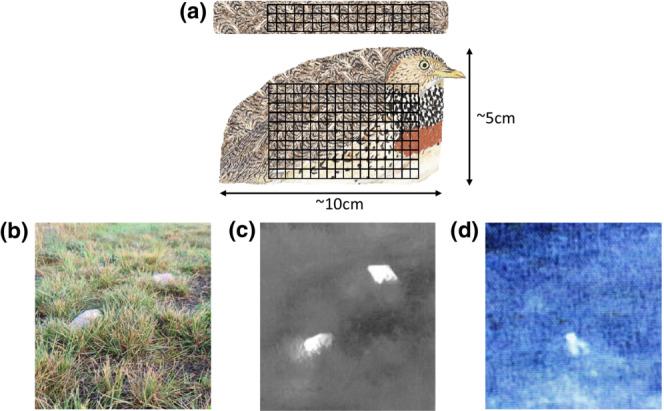
Models of Plains‐wanderers (*P. torquatus*), artificially heated with heat packs (placed inside the models) and insulated with a cardboard base, simulating small endotherms for detection with thermal scanners and spotlighting methods. (a) Plains‐wanderer model design and dimensions with heat pack position (represented by crosshatch) viewed from the top (top) and the side (bottom); (b) appearance of models during daylight; (c) appearance of models at night with the aid of a thermal scanner; and (d) appearance of a Plains‐wanderer at night with the aid of a thermal camera. Heated models maintained a temperature of 33.6–44.3°C for ~5 h in uncontrolled temperatures.

Six independent 200 m‐long transects with fixed 40 m strip widths (20 m each side of a centrally‐located transect) were established within Plains‐wanderer habitat (Nugent et al., 2022). A random number (range = 20–40) of Plains‐wanderer models were positioned following a computer‐generated random array along each transect by people who were not observers in the study. Models were placed at random distances along the transect and random distances perpendicular to the transect. Plains‐wanderer models (Figure 2; Appendix 2) were artificially heated by placing a heat pack (charcoal activated Little Hotties® Hand Warmers) inside the model, which maintained a temperature of 33.6–44.3°C for ~5 h in uncontrolled temperatures (Appendix 3—Figure A2). The models were visually representative of Plains‐wanderers under spotlights because Plains‐wanderers do not have eyeshine and the models were carefully designed and placed to mimic a Plains‐wanderer sitting in a grassland at night (Figure 2a,b). The models were visually representative of Plains‐wanderers via thermal cameras due to heat packs maintaining the body temperature of an endotherm, with the temperature variations in heat packs mimicking the natural variation you would observe in endotherms caused by differences in insulation by fur, feathers, or hair (Figure 2c,d).

Surveys were conducted by five observers who had no prior knowledge of the number, strip width, and array of heated models. Observers surveyed each transect twice employing either a 1300 lumen spotlight or one of three thermal scanners (Table 1, see Appendix 4—Table A1 for balanced survey design). Observers traversed transects in the opposite direction on their second survey, and did so after traversing another independent transect, to minimize the possibility that specific locations of models would be recalled. In total, 60 surveys were performed across all transects, such that a cumulative transect length of 12 km was traversed on foot by observers with the opportunity to detect 1800 artificial models.

**TABLE 1:** Specifications of handheld thermal scanners assessed in this study, which represent the range of portable, handheld thermal scanners currently available to practitioners.

	Low‐resolution scope	Medium‐resolution scanner	High‐resolution scope
Model	Scout TK, FLIR Systems Inc., North Billerica, MA, USA	InfReC G120EX, Nippon Avionics Co., LTD., Tokyo, Japan	Pulsar Helion XP28, Yukon Advanced Optics Worldwide, LTD., Vilnius, Lithuania
Body type	Scope (single‐eye)	Video camera	Scope (single‐eye)
Spectral range (μm)	7.5–13.5	8–14	8–14
Field of view (°)	20 × 16	32 × 24	22 × 16.6
Resolution (microbolometer)	160 × 120	320 × 240	640 × 480
Thermal sensitivity (NETD at 30°C)	0.06°	0.04°	0.06°
Purchase price ($AUD)	$1000	$9800	$6000

### 2.3 Statistical methods

Detection probability curves were generated for each device in the ‘Distance’ R package (Miller, 2016) by splitting the data set and fitting distance sampling models for each device separately. Hazard‐rate and half‐normal key functions were considered for each model, and the best‐fitting unadjusted detection functions were selected based on lowest Akaike's information criterion (AIC; Buckland et al., 1993). The covariate ‘observer’ was included where appropriate to achieve best‐fit models for each device.

To measure the effect of device on detection rates (calculated as percentage of available models that were detected during a survey), a linear mixed‐effects (LME) model (package ‘nlme’, Pinheiro et al., 2020) with post hoc pairwise comparisons (package ‘emmeans’, Lenth et al., 2020) was conducted with observer and transect included as random effects. The LME model assumption of normality was checked by visually inspecting QQ plots and was met.

Accuracy, defined as the closeness of an estimate to the true number, was assessed by the modeled abundance of heated models in the sampled area (according to distance sampling models) divided by the true number of models (Hodgson et al., 2016; Sorrell et al., 2019). Precision, defined as the closeness of replicated estimates to one another, was assessed using a Levene's Test (package ‘car’, Fox & Weisberg, 2019) to test for difference in variance (standard error) from the LME model of detection rate from each device (Hodgson et al., 2016; Sorrell et al., 2019). All data analyses were undertaken in R Studio version 1.1.463 (RStudio Team, 2020).

### 2.4 Written usability survey

Following field surveys, a written survey was completed by each of the five observers. Seven written survey questions explored the observers' experiences using each survey device, focusing on ease of use and comfort throughout numerous hours of field surveys (see Appendix 5—Table A2 for full survey questions). Observers answered each question with a score of 1 (strongly disagree) to 5 (strongly agree).

## 3 RESULTS

### 3.1 Detection rates

**FIGURE 3:**
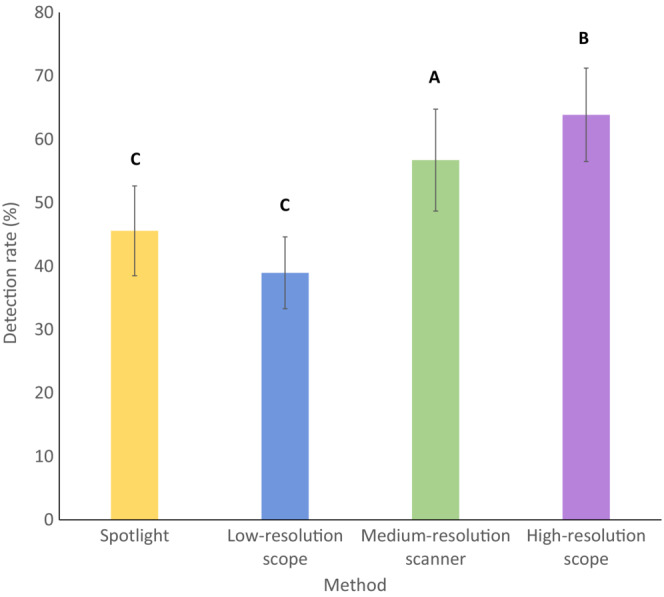
Observed detection rate (mean percentage of models detected) and standard error of three handheld thermal scanners and a spotlight during night‐time surveys of simulated populations of artificially heated Plains‐wanderers (*P. torquatus*) along 200 m long transects in native Victorian grasslands. Letters have been added to bars to denote statistically significant differences between tools. Tools that share letters are not significantly different in detection rate.

In total, 924 of a possible 1800 detections of artificially heated models were made across all survey methods. Detection rate differed significantly between survey methods (*F*_3, 27_ = 17.98, *p* < .001). The high‐resolution scope (mean 63.85% of models detected per survey, *n* = 293) demonstrated significantly greater detection rates than the medium‐resolution scanner (mean 56.71% of models detected, *n* = 249, β = 8.44, SE = 3.46, *p* = .043), the low‐resolution scope (mean 38.95% of models detected, *n* = 180, β = 23.22, SE = 3.28, *p* < .001), and the spotlight (mean 45.58% of models detected, *n* = 202, β = 18.35, SE = 3.71, *p* < .001). The medium‐resolution scanner showed significantly greater detection rate than the low‐resolution scope (β = 14.78, SE = 3.71, *p* = .006) and the spotlight (β = 9.91, SE = 3.28, *p* = .017; Figure 3).

### 3.2 Probability of detection

**FIGURE 4:**
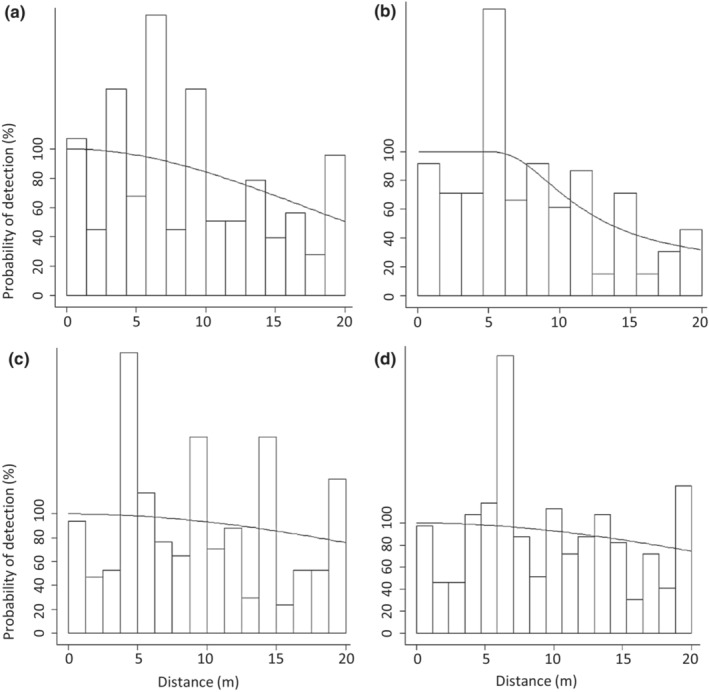
Estimated probability of detection of artificially heated Plains‐wanderer (*P. torqautus*) models over a distance of 20 m for four devices: (a) spotlight; (b) low‐resolution thermal scope; (c) medium‐resolution thermal scanner; and (d) high‐resolution thermal scope. Histograms represent actual numbers of detections and fitted lines represent probability of detection.

Detection probabilities were fitted with the best‐fit models according to lowest AIC values (Table 2). All devices provided observers with near‐perfect detection for the first ~5 m. Over the full 20 m distance, the high‐resolution scope and medium‐resolution scanner maintained the highest detection probabilities (declining to ~75% and ~77%, respectively; Figure 4a,b). By contrast, detection probability declined more abruptly at >5 m from the observer for the low‐resolution scope and the spotlight to ~37% and ~48%, respectively, for objects at 20 m (Figure 4c,d).

**TABLE 2:** Distance sampling models and accuracy of devices used during night‐time surveys to detect artificially heated Plains‐wanderer models in native Victorian grassland.

Device	Best fit model (key function and covariates)	Number of detections	Modeled abundance	True number of models	Accuracy (modeled abundance/true number)
Spotlight	Unadjusted half normal, no covariates	202	248.80 (±18.17)	442	0.56
Low‐resolution Thermal Scope	Hazard rate, covariate = observer	180	254.90 (±21.27)	458	0.56
High‐resolution Thermal Scope	Unadjusted half normal, no covariates	292	313.55 (±19.76)	462	0.68
Medium‐resolution thermal scanner	Unadjusted half normal, no covariates	249	272.03 (±18.52)	438	0.62

*Note*: Modeled abundance (± standard error) of models is based on distance sampling.

Effective strip widths (distance at which the detected number of objects found up to that point are equal to the number that are estimated to occur beyond) were found to be 14.12 m for the low‐resolution scope and 16.24 m for the spotlight, compared to 18.63 m for the high‐resolution scope and 18.31 m for the medium‐resolution scanner.

### 3.3 Accuracy and precision

The high‐resolution scope and the medium‐resolution scanner had the highest accuracy, represented by modeled abundances that were closest to the true number of models present during surveys. The spotlight and low‐resolution scope conferred lower survey accuracy (Table 2). Precision was not significantly different between the devices, such that the consistency of estimates between surveys was not significantly different between devices (*F*_3,56_ = 0.340, *p* = .797).

### 3.4 Written usability survey

Observers found the high‐resolution scope and medium‐resolution scanner to be overall the most usable and suitable for night‐time surveys for detecting small endotherms (Table 3, see Appendix 5—Table A2 for full survey results). Spotlights were easy to use and allowed observers to easily follow the transect line, and as such observers considered them the easiest device to conduct surveys with. Observers found they could readily detect and clearly see the models with all devices other than the low‐resolution scope. The low‐resolution scope was also considered the least ergonomic when being handheld throughout surveys, and observers required more short breaks during surveys due to physical discomfort, particularly eye fatigue.

**TABLE 3:** Scores from a written usability survey completed by five observers that participated in night‐time surveys using four devices to detect artificially heated Plains‐wanderer models in native Victorian grassland.

Survey category	Spotlight	Low‐resolution thermal scope	High‐resolution thermal scope	Medium‐resolution thermal scanner
Ease of conducting surveys	50	33	44	40
Ease of detection	42	24	47	45
Physical comfort	39	31	41	42
Suitability for detecting small endotherms	15	6	25	23
Total	146	94	157	150

*Note*: Responses were provided as a score out of 5 (1 = strongly disagree, 2 = disagree, 3 = neutral, 4 = agree, 5 = strongly agree). The three categories dealing with ease of use and ergonomics each consisted of two questions (maximum available score of 50), the last category assessing suitability for target endotherms consisted of a single question (maximum available score of 25). The total available score for each device was 175.

## 4 DISCUSSION

This study presents an experimental comparison of multiple thermal scanners against traditional spotlights as survey tools for detecting small endotherms at night. As thermal scanners emerge as a wildlife monitoring tool, studies like ours ensure that accurate data on the capabilities of thermal scanners are available to scientists and wildlife managers. In particular, our study provides valuable insight into the capabilities of thermal scanners for monitoring small endotherms in open grasslands compared to the traditional method of spotlighting.

Considering all measures of performance (detection rate, detection distance, accuracy, and precision), the high‐ and medium‐resolution thermal scanners performed the best. Detection rates, detection probability, and survey accuracy were higher for high‐ and medium‐resolution thermal scanners than for the low‐resolution thermal scope and spotlight. Although the transect width was limited to 40 m total, and the tails of the distance models (beyond 20 m distance) could not be estimated, the sampled strip width provided a clear comparison between methods and understanding of tool capabilities. Observer experience suggested that spotlights provided adequate ease of detection, performance measures show that high‐ and medium‐resolution thermal scanners provided a significant improvement in detection capabilities over the traditional method of spotlighting. This highlights the value of experimental comparisons when determining the performance of different survey methods.

The clear image provided by high‐ and medium‐resolution thermal scanners allowed the model Plains‐wanderers to be easily differentiated from other relatively warm objects (e.g., rocks, grass tussocks). McGregor et al. (2021) highlighted the challenge of differentiating between warm non‐target objects and endotherms when using thermal scanners in warm environments (>30°C), suggesting that night‐time use of thermal scanners is best suited to cooler months (McGregor et al., 2021). In addition, there was a consensus among users that the high and medium‐resolution scanners were more comfortable to hold and view than the low‐resolution scanner while traversing the transect. This in part arises because prolonged use typical of fauna surveys can result in considerable eye fatigue; when a scope is held to an observer's eye, that pupil contracts whilst the other dilates in a nocturnal setting, and this fatigue is exacerbated by low‐resolution imagery. The low‐resolution thermal scope is not an appropriate tool for detecting small endotherms because the low‐resolution image did not allow users to readily detect small objects and caused eye fatigue.

The distance at which observers had 50% detection probability in this study could only be determined for the low‐resolution scope (~14 m), and this was lower than the theoretical maximum detection distance using Johnson's detection criteria (~28 m; Figures 1 and 4). This demonstrates that in field settings, background clutter impacts an observer's ability to distinguish warm objects, and any vegetation can obscure line of sight—especially when searching for small objects. Cluttered environments are known to interfere with object detection when using thermal scanners for the detection of vehicles and humans (Alkandri et al., 2011; Brunnstroem et al., 2003). If a wider transect was sampled, Johnson's detection criteria could be used to better understand the effect of background clutter when using different methods. According to the usability survey, observers were more likely to mis‐identify a warm object as a Plains‐wanderer model while on the transect when using the low‐resolution scope compared to the medium‐ and high‐resolution scanners. This indicates that higher resolution thermal scanners improve an observer's ability to distinguish between warm objects. Background clutter and vegetation density relative to the size of the target species should be accounted for when considering thermal scanners for detection and monitoring of threatened species.

Field of view is also likely to have impacted the detection capabilities of devices in this study, as it determines the size of the area that can be surveyed (although the very broad field of view produced by fish‐eye lenses should be avoided as they cause distortion; Cilulko et al., 2013; Steen et al., 2012). Johnson's detection criteria predicted that the high‐resolution scope would confer a higher detection probability than the medium‐resolution scanner; however, the detection probability over the 20 m transect width was largely similar for these two devices. The high‐resolution scope had a narrower field of view than the medium‐resolution scanner, which likely reduced its detection rate by restricting users' ability to thoroughly scan the full width of the transect.

We recommend that thermal scanners with resolutions ≥320 × 240 be implemented as ‘best practice’ for night‐time surveys of small endotherms. We also conclude that scanners with a separate display are superior to scopes in terms of usability. Thermal scanners will be a particularly important tool in the monitoring and management of threatened small endotherms where detection of individuals is inhibited by crypsis, nocturnal activity patterns, avoidance behaviors and low abundance. We expect these tools can be used to study and monitor a number of species in open environments where it was previously extremely difficult or impossible, including the Plains‐wanderer, Fat‐tailed Dunnart (*Sminthopsis crassicaudata*) and Bengal Florican (*Houbaropsis bengalensis*) in grasslands, Julia Creek Dunnart (*S. douglasi*) in savanna, Kowari (*Dasyuroides byrnei*) in gibber plains, and the Eastern and Western Ground Parrots (*Pezoporus wallicus* and *P. flaviventris*) in grasslands and shrublands (Birdlife International, 2016, 2018a, 2018b; Burbidge et al., 2016; Burnett & Winter, 2019; McKnight et al., 2019). Such species have been notoriously difficult to detect with exceedingly low detection rates using spotlights. For example, detection rates of Plains‐wanderers during dedicated monitoring surveys range from 0.015 to 0.3 birds/km (Baker‐Gabb et al., 2016). Here we demonstrate that by employing an appropriate thermal scanner during night‐time surveys of Plains‐wanderer models, significant increases in detection rate and accuracy can be achieved when compared with spotlighting. Given our models were closely representative of a small endotherm, thermal scanners can improve practitioners' ability to effectively monitor and manage populations of small endotherms in open grasslands where this was previously hindered or not possible due to extremely low detection rates.

## AUTHOR CONTRIBUTIONS

**Finella M. E. Dawlings:** Data curation (lead); formal analysis (lead); funding acquisition (supporting); investigation (equal); project administration (lead); validation (equal); visualization (lead); writing – original draft (lead); writing – review and editing (equal). **Claire Mackay:** Conceptualization (supporting); data curation (supporting); formal analysis (supporting); investigation (equal); methodology (equal); project administration (supporting); validation (supporting); writing – review and editing (equal). **Morgan Humphrey:** Investigation (equal); methodology (supporting); writing – review and editing (equal). **William F. Mitchell:** Investigation (equal); writing – review and editing (equal). **Karina J. Sorrell:** Investigation (equal); writing – review and editing (equal). **Sonia Sanchez:** Investigation (equal); writing – review and editing (equal). **Benjamin M. Viola:** Investigation (equal); methodology (supporting); project administration (supporting); writing – review and editing (equal). **Rohan H. Clarke:** Conceptualization (lead); funding acquisition (lead); investigation (equal); methodology (equal); project administration (supporting); resources (lead); supervision (lead); validation (equal); visualization (supporting); writing – original draft (supporting); writing – review and editing (equal).

## FUNDING INFORMATION

None.

## CONFLICT OF INTEREST STATEMENT

The authors declare no conflict of interest associated with this manuscript.

## Figures and Tables

**FIGURE A1:**
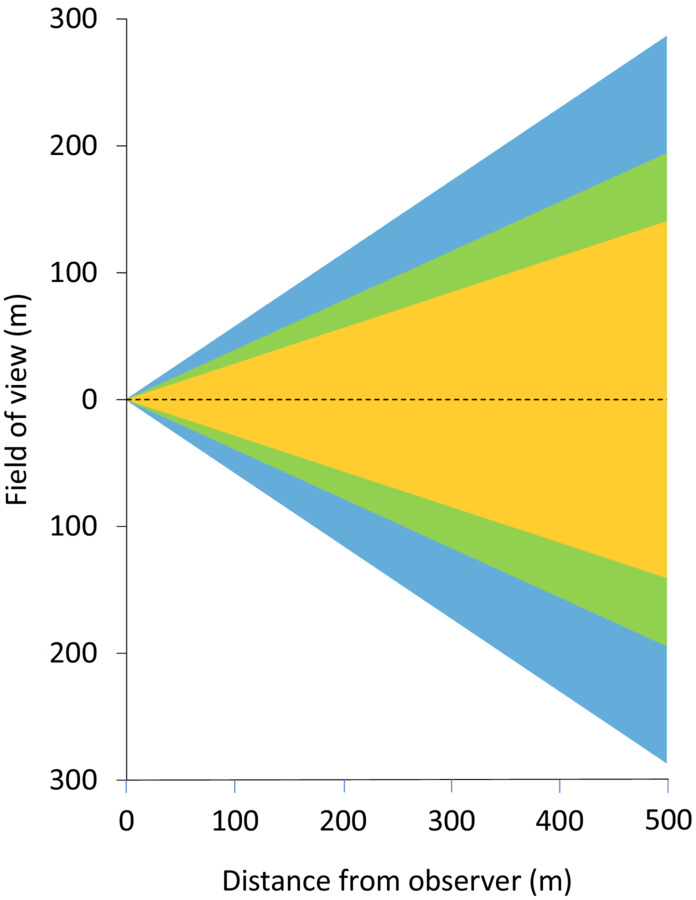
Field of view at 0–500 m from the observer for handheld thermal cameras with three standard horizontal angles of view; 16° (yellow), 22° (green) and 32° (blue).

**FIGURE A2:**
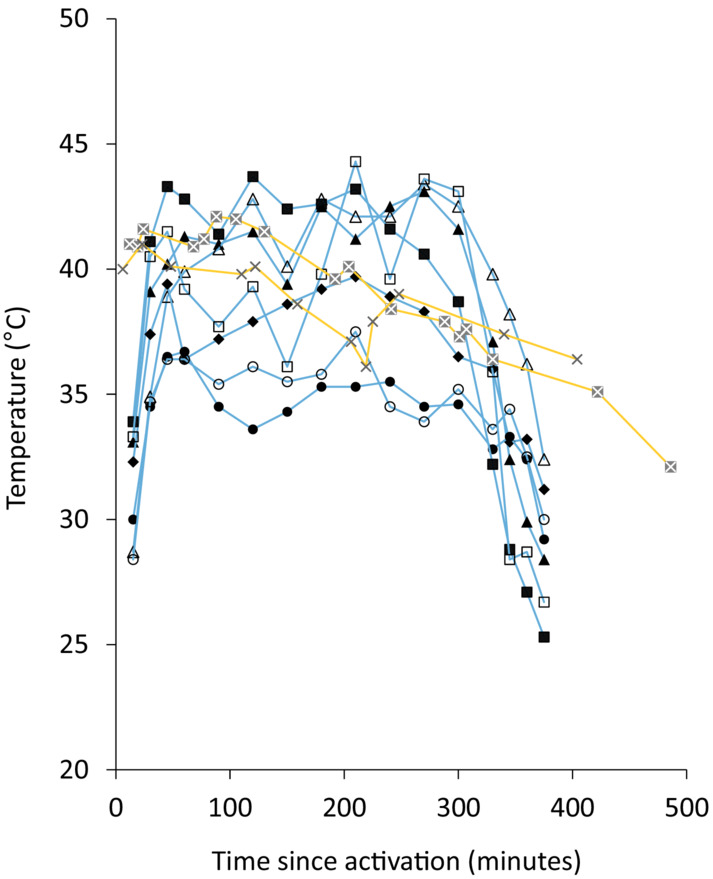
Temperature of Little Hotties® Hand Warmers following activation. Seven hand warmers were placed in uncontrolled settings (blue) with ambient temperature ranges of 9.4–15.7°C, and two were placed in a controlled setting (yellow) where ambient temperatures remained stable at ~23°C.

## Data Availability

Data available from Monash University's BRIDGES account. doi:10.26180/23626311.v1.

## APPENDIX 2

### CONSTRUCTION OF ARTIFICIALLY HEATED PLAINS‐WANDERER MODELS

Model templates of Plains‐wanderer (*Pedionomus torquatus*) were created in Adobe Photoshop CS6 (Adobe Systems, Inc.) using illustrated plates from ‘The Australian Bird Guide’ (Menkhorst et al., 2017). Hollow, non‐reflective polyethylene models were artificially heated with Little Hotties® Hand Warmers (Implus Footcare, LLC) and insulated with a cardboard base.

## APPENDIX 3

### TESTING OF ARTIFICIALLY HEATED PLAINS‐WANDERER MODELS

To determine the suitability of Little Hotties® Hand Warmers to simulate the heat of a Plains‐wanderer (*P. torquatus*), the maximum temperature range and duration of time that they were able to maintain heat was assessed in controlled and uncontrolled settings. Heated models could be detected with the handheld thermal scanner and high‐resolution thermal scope at a maximum distance of 20 m, and were not reflective under torchlight. Heated models maintained a temperature range of 33.6–44.3°C in uncontrolled and controlled settings for ~5 and ~8 h, respectively (Figures A1 and A2). These stable temperatures appropriately represent the heat of a small endotherm.

## APPENDIX 4

### BALANCED SURVEY DESIGN

**TABLE A1:** Number of surveys performed across six transects with four survey devices during night‐time surveys to detect artificially heated Plains‐wanderer (*P. torquatus*) models in native Victorian grasslands, where five observers surveyed each transect twice.

Transect	Spotlight	Low‐resolution thermal scope	High‐resolution thermal scope	Handheld thermal scanner	Total number of surveys
1	2	3	2	3	10
2	3	2	3	2	10
3	2	3	3	2	10
4	3	2	2	3	10
5	3	2	2	3	10
6	2	3	3	2	10
Total number of surveys	15	15	15	15	60

## APPENDIX 5

### WRITTEN USABILITY SURVEY

**TABLE A2:** Scores from a written usability survey completed by five observers that participated in night‐time surveys using four devices to detect artificially heated Plains‐wanderer (*P. torquatus*) models in native Victorian grassland.

Survey questions	Spotlight	Low‐resolution thermal scope	High‐resolution thermal scope	Handheld thermal scanner
I was able to easily follow the transect for the whole survey, and I did not lose track of the transect line	25	10	20	22
When I detected a model Plains‐wanderer within 5 m of the transect line, I could see it clearly with the device	23	14	25	25
When I detected a Plains‐wanderer model with the device and walked out to check, it was always a Plains‐wanderer model rather than another, mis‐identified object	19	10	22	20
I found the device comfortable to hold when in use	23	17	20	22
I did not have to take breaks during surveys due to the device	16	14	21	20
I was able to quickly and easily learn how to use the device	25	23	24	18
I would recommend this device for ecological surveys targeting small endotherms	15	6	25	23
Total	146	94	157	150

*Note*: Answers were given as a score out of 5 (1 = strongly disagree, 3 = neutral, 5 = strongly agree), such that each score is out of 25, and the total score for each device is out of 175.
